# Image-Guided Minimally Invasive Treatment Options for Degenerative Lumbar Spine Disease: A Practical Overview of Current Possibilities

**DOI:** 10.3390/diagnostics14111147

**Published:** 2024-05-30

**Authors:** Makoto Taninokuchi Tomassoni, Lorenzo Braccischi, Mattia Russo, Francesco Adduci, Davide Calautti, Marco Girolami, Fabio Vita, Alberto Ruffilli, Marco Manzetti, Federico Ponti, George R. Matcuk, Cristina Mosconi, Luigi Cirillo, Marco Miceli, Paolo Spinnato

**Affiliations:** 1 Diagnostic and Interventional Radiology, IRCCS Istituto Ortopedico Rizzoli, 40136 Bologna, Italy; 2 Radiology Department, IRCCS Azienda Ospedaliero-Universitaria Sant’Orsola Malpighi, 40138 Bologna, Italy; 3 Neuroradiology, IRCCS Istituto delle Scienze Neurologiche di Bologna, 40139 Bologna, Italy; 4 Spine Surgery Unit, IRCCS Istituto Ortopedico Rizzoli, 40136 Bologna, Italy; 5 1st Orthopaedic and Traumatologic Clinic, IRCCS Istituto Ortopedico Rizzoli, 40136 Bologna, Italy; 6 Department of Imaging, Cedars-Sinai Medical Center, Los Angeles, CA 90048, USA

**Keywords:** low back pain, pain management, lumbar osteoarthritis, intervertebral disc degeneration, interventional ultrasonography, interventional radiology, magnetic resonance imaging, multidetector computed tomography, pulsed radiofrequency treatment

## Abstract

Lumbar back pain is one of the main causes of disability around the world. Most patients will complain of back pain at least once in their lifetime. The degenerative spine is considered the main cause and is extremely common in the elderly population. Consequently, treatment-related costs are a major burden to the healthcare system in developed and undeveloped countries. After the failure of conservative treatments or to avoid daily chronic drug intake, invasive treatments should be suggested. In a world where many patients reject surgery and prefer minimally invasive procedures, interventional radiology is pivotal in pain management and could represent a bridge between medical therapy and surgical treatment. We herein report the different image-guided procedures that can be used to manage degenerative spine-related low back pain. Particularly, we will focus on indications, different techniques, and treatment outcomes reported in the literature. This literature review focuses on the different minimally invasive percutaneous treatments currently available, underlining the central role of radiologists having the capability to use high-end imaging technology for diagnosis and subsequent treatment, allowing a global approach, reducing unnecessary surgeries and prolonged pain-reliever drug intake with their consequent related complications, improving patients’ quality of life, and reducing the economic burden.

## 1. Introduction

### 1.1. Epidemiology and Economic Impact

Lumbar back pain (LBP) is one of the most common presenting symptoms of patients worldwide. During their lifetime, 80% of Americans will experience at least one episode of LBP [1,2,3]. LBP is also considered the leading cause of years lived with disability around the world, and sixth in terms of overall disease burden (disability-adjusted life years) [4,5,6]. The economic impact to healthcare services is another substantial LBP-related problem, having a total cost of more than USD 100 billion per year in the United States [7]. The same problem is presented in European countries, with Germany arriving at a total cost of EUR 48.96 billion per year [8].

Although it has been documented that 90% of LBP cases are usually resolved within 1 month [9], Hestbaek et al. [10] found a high recurrence rate, concluding that generally between 44 and 78% of patients will experience relapse.

LBP can be divided into acute (lasting less than 6 weeks), subacute (6–12 weeks), or chronic (more than 12 weeks) according to the duration of symptoms, and can also be divided into non-specific/specific depending on its cause [11].

### 1.2. Pathogenesis and Main Causes

Multiple lumbar structures are considered plausible pain generators, including the intervertebral disc and vertebral facet joints (FJ) [2,12]. Facet joint syndrome (FJS) refers to unilateral or bilateral back pain that radiates caudally through one or both buttocks, sides of the groin, and thighs, and usually stops above the knee [13]. The principal cause of FJS is FJ osteoarthritis, which consists of joint space narrowing/cartilage loss, ligamentum flavum thickening, synovial fluid/synovitis, and bony over-growth (osteophytes). All of these features can lead to chronic inflammation and, consequently, LBP [14]. FJS may be considered the cause of LBP in 10–15% of young patients and plausibly in a higher percentage of the elderly population [15]. On the other hand, degenerative disc disease (DDD) refers to an intervertebral disc structure pathological condition that is also associated with LBP. Extracellular matrix composition alteration related to a decrease in disc nutrients and oxygen supply can lead to impaired disc function [16,17]. Plausible risk factors for disc degeneration include genetics, obesity, cigarette use, and cell senescence [18,19,20,21,22,23]. Other causes include spine instability [24] and spinal epidural lipomatosis [25].

Regarding specific types of pain, radicular pain needs a special mention. Also known as radiculopathy, this is a type of pain that radiates along the nerve path, often stemming from irritation, compression, or inflammation of the spinal nerve roots. This condition typically occurs when the spinal nerves, which extend from the spinal cord to various parts of the body, become compromised or compressed. The resulting pain can be sharp, shooting, or burning, and may be accompanied by tingling, numbness, or weakness along the affected nerve pathway. Multiple causes of this kind of pain have been reported, such as herniated discs and other types of DDD, spinal stenosis, and neural foraminal narrowing [26].

### 1.3. Indication for Surgery and Conservative Treatments

There are several treatment options for LBP, including behavioral therapy, exercise, multidisciplinary rehabilitation, anti-inflammatory drug administration, minimally invasive image-guided treatments, and, finally, surgery [27]. As a starting point, patient counseling is an integral part of care, including information on LBP pathology, and is designed to encourage the patient to be physically active and continue with normal activities [28].

In terms of drug treatment, a recent systematic review of clinical guidelines for the management of LBP recommends non-steroid anti-inflammatory drugs (NSAIDs) to relieve short-term symptoms in patients with acute LBP. In cases of persistent pain or contraindications for NSAIDs, weak opioids and oral corticosteroids can be used for short-term management. However, strong opioid drugs for LBP are not recommended [29].

Physical therapy is the key element in the treatment of LBP, especially in the chronic phase, reducing pain and improving function. A study by Hayden et al. highlighted how low-load programs are useful for relieving LBP [30]. In recent years, there has been much emphasis on multidisciplinary and behavioral rehabilitation as an essential part of initial conservative treatment. Indeed, the study of Lambee et al. [31] demonstrates how multidisciplinary rehabilitation is effective and increases workplace reintegration.

Surgery is an option for patients with DDD who are unresponsive to conservative treatment. Spinal claudication symptoms indicative of spinal canal stenosis and neural deficit are above all the main clinical indications for spinal surgery. Instability is another indication, mostly occurring in combination with some degree of spinal stenosis, with a dynamic component of worsening under mechanical load (and warranting a fusion procedure). Chronic back pain for more than 6 months, with or without radiation, is also considered an indication [32]. A conservative treatment program should be followed for at least 1 year before considering surgery for non-specific low back pain [33]. However, in case of specific low back pain, depending upon the relevance and severity of radiological findings, its progressive nature, and neurological status, conservative management could be shorter [34].

In the last few years, spine surgery has witnessed several innovative approaches aimed at addressing low back pain and associated spinal conditions. Lumbar interbody fusion (LIF) techniques have evolved significantly, offering diverse approaches for treating low back pain and related conditions. Posterior lumbar interbody fusion (PLIF) involves accessing the spine through the back using a midline approach, removing disc material and fusing vertebrae using bone grafts and implants. Anterior lumbar interbody fusion (ALIF) accesses the spine through the abdomen, allowing for disc removal and fusion from the front, reducing the risk of nerve damage and facilitating spinal sagittal alignment correction. Laterally, both oblique (OLIF) and direct (DLIF, also known as extreme lateral interbody fusion [XLIF]) approaches offer minimally invasive access to the disc space through the side, sparing posterior muscles and nerves while achieving fusion. Often, OLIF and XLIF are combined with posterior stabilization to achieve circumferential fusion. Transforaminal lumbar interbody fusion (TLIF) is another posterior surgery where the cage is inserted through a para-median approach to avoid retracting the thecal sac and nerve roots. Additionally, extreme lateral interbody fusion (XLIF) accesses the spine laterally, reducing muscle disruption and enhancing recovery [35].

While all approaches give good results, multiple studies have shown that XLIF and OLIF offer better outcomes over the other techniques in terms of blood loss, lower sympathectomy risk, shorter hospital stays, and lower subsidence rates. Recently, some studies also demonstrated that OLIF has fewer neuromuscular complications compared with XLIF [36,37,38,39,40,41,42,43]. 

## 2. Image-Guided Minimally Invasive Treatments

Image-guided procedures are considered minimally invasive treatments for LBP, after failure of conservative therapy [44]. Overall, minimally invasive treatments are far less expensive than surgery and produce good results, as demonstrated in some meta-analyses [45,46,47].

Additionally, there is a very low incidence of adverse effects (average complication rate is less than 0.5%). These rare complications are mostly related to intradiscal procedures, including spondylodiscitis, allergic reactions, disc collapse, hemorrhages, and, rarely, neural injuries [44,48]. Disc degeneration directly correlated to an intradiscal injection is extremely rare [48].

### 2.1. Patient Preparation and Management

**Figure 1:**
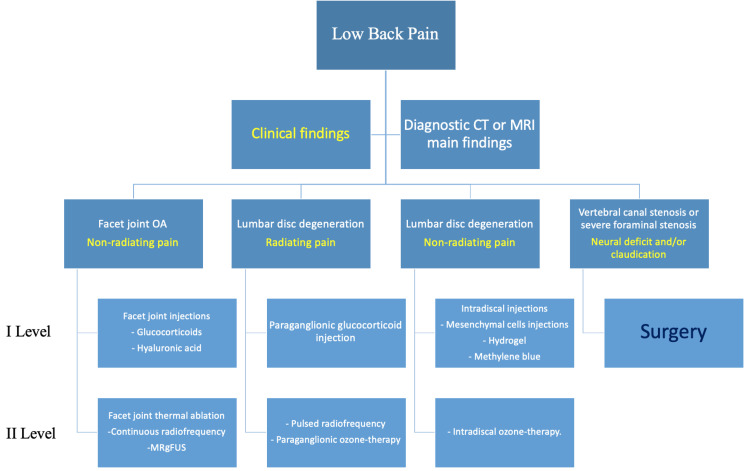
Proposed flowchart for management of patients with degenerative lumbar pain. Main Clinical findings (yellow).

Every patient should always be evaluated through physical examination and diagnostic imaging techniques, such as computed tomography (CT) or magnetic resonance imaging (MRI), and their case preferably discussed in a multidisciplinary environment (orthopedic surgeons, radiologists, and physiatrists) to decide the correct imaging method and most suitable treatment (Figure 1).

MRI is considered the most sensitive and comprehensive imaging tool available for the study of the lumbar spine, providing information in regard to all the structures involved in the pathogenesis of low back pain, including bone, disc, neural roots, facet joints, ligaments, paravertebral muscles, spinal canal, neural foramina, and epidural fat. Several MRI severity-grading systems have been proposed to help clinicians in the management of patients with low back pain. Most of them are focused on spinal canal narrowing, with a few others also including grading of the lateral foramen. Recently, Spinnato et al. provided a comprehensive MRI severity-grading system for central and lateral lumbar spine stenosis, with inclusion of the main causes of disease schematized into four categories: (i) disc, (ii) arthritis, (iii) epidural lipomatosis, and (iv) mixed causes [49]. This is currently the most comprehensive and the only system to include all causes of stenosis, and can rapidly help clinicians to choose the appropriate treatment strategies, including surgery, image-guided minimally invasive treatments, and conservative treatments (e.g., oral drugs and weight loss). Including epidural lipomatosis among the main causes of stenosis with disc pathology and arthritis is an important and unique feature of this grading system. Recent literature has recognized that hypertrophy of epidural fat is one of the key elements in the development of symptoms and lumbar stenosis [50,51,52].

There is no universal agreement on sedation, local anesthetic, or antibiotic prophylaxis for preoperative care [53]. Some authors recommend not using local or general anesthesia because they could disguise nerve root puncture symptoms [48]. 

The choice of the correct imaging guidance should be carefully evaluated according to:

(i) Target anatomical site of injection;
(ii) Patient’s age and body mass index;
(iii) Type of treatment;
(iv) Operator’s experience.

In Table 1, we provide a summary of the different imaging modalities available for guidance of treatments, along with their advantages and disadvantages, and their main indications.

### 2.2. Image-Guided Approaches

We established three image-guided approaches and two levels of treatment, based on complexity and cost. The technique is linked to LBP etiology.

#### 2.2.1. Intradiscal Approach

Discogenic issues have been reported to account for 40% of all LBP causes [48]. Heating (e.g., radiofrequency, microwave, and laser) or the injection of different chemicals into the intervertebral disc may alter the internal mechanics of the disc and alleviate neuropathic pain by re-creating the intervertebral disc’s form and structure [54,55]. The secondary aim is to decrease the pressure within the spinal canal, which releases the pressure on the patient’s nerve roots and minimizes their clinical symptoms [55].

With fluoroscopy, angulation refers to the tilting or angling of the X-ray tube and image receptor to visualize specific anatomical structures from different perspectives. Craniocaudal inclination involves angling the X-ray tube and detector vertically to visualize structures along the head-to-toe axis. Lateral inclination entails angling the equipment horizontally to visualize structures from side to side. These adjustments are crucial for obtaining optimal views of anatomical structures and aiding in the accurate diagnosis and treatment of various medical conditions during real-time imaging procedures.

**Figure 2:**
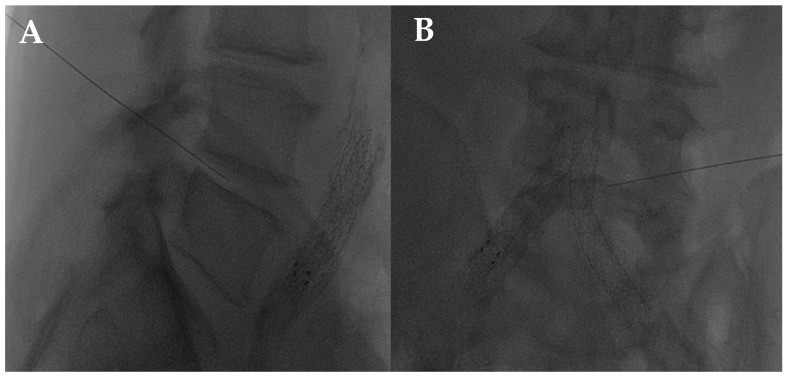
(**A**,**B**) Lateral and frontal view on fluoroscopic images of a lumbar intersomatic disc puncture with a 22G needle.

**Figure 3:**
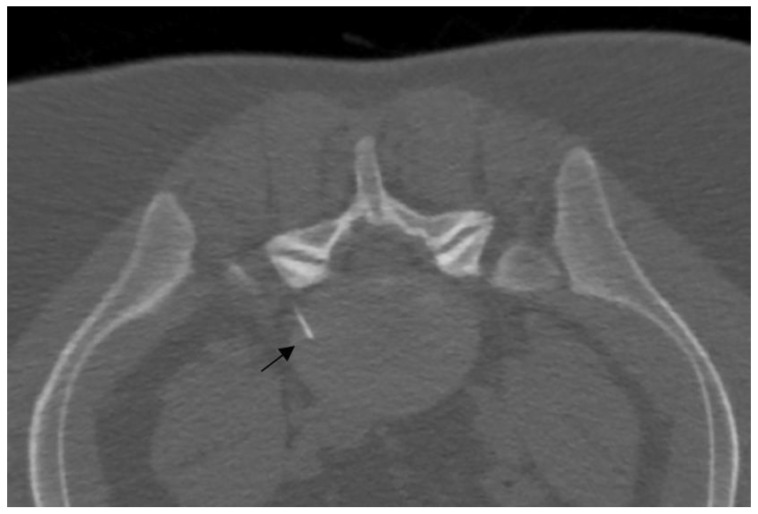
A 25-year-old patient with L5 discogenic pain treated with ozone therapy; notice the tip of the needle is located in the lumbar disc (black arrow).

The disc center is the ideal injection target, and the prone position, employing support under the abdomen or the neck to increase the posterior vertebral space, is the optimum posture to expand the intervertebral space [48]. The access site is always selected after radiographic evaluation of the needle path; it is suggested to follow a lateral inclination of 45° to 60°, with an additional craniocaudal inclination for lumbar discs [56,57]. Some authors indicate the foramen radicularis, maintaining the root laterally and superiorly, as the best way to access the center of the disc (Figure 2 and Figure 3) [58].

#### 2.2.2. Transforaminal Approach

**Figure 4:**
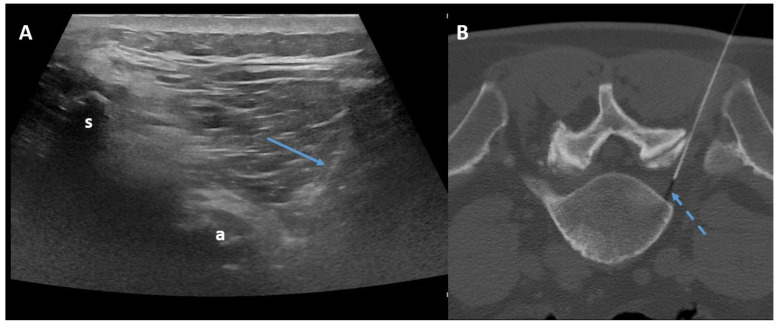
Ultrasound-guided (**Panel A**) and CT-guided (**Panel B**) lumbar paraganglionic drug injection. Blue arrow = needle visualized on ultrasound. Dotted blue arrow = ganglion root. s = spinous process, a = posterior articular complex.

The transforaminal approach permits access to the compressed nerve roots that are a plausible origin of pain. The prone position with support under the abdomen or under the neck is sufficient both for the lumbar spine and for the cervical spine. Fluoroscopy, CT, or ultrasonography (US) may guide the needle, helping to maintain an inclination of 45° to 60°, laterally to the pedicles (Figure 4).

**Figure 5:**
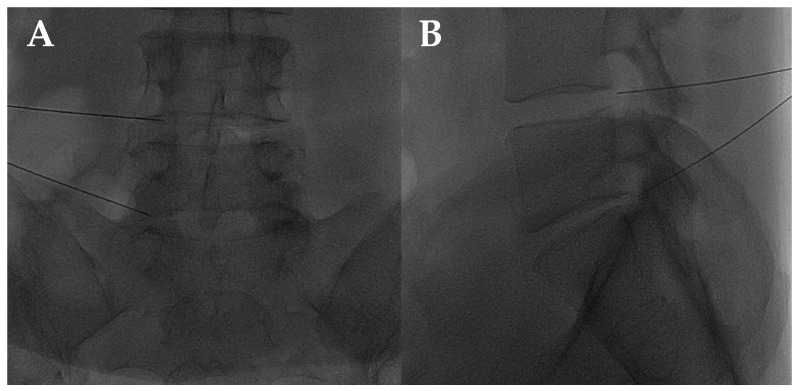
(**A**,**B**) Frontal and lateral view on fluoroscopic images of lumbar transforaminal injections with 22G needles.

In fluoroscopy, the C-arm may be rotated to an angle of 45° along the same direction of the treated side, producing a superimposed virtual triangle, called the Scotty dog appearance, which guides the needle trajectory [59]. On CT, axial scans easily help to monitor the path from skin to foramen. The target is the foramen radicularis, and root contact is unnecessary. Radiopaque dyes can be used in order to confirm the needle placement if necessary. Therefore, once close to the nerve, medications may be injected, and the needle can be removed (Figure 5) [58,59].

#### 2.2.3. Facet Joint Approach

**Figure 6:**
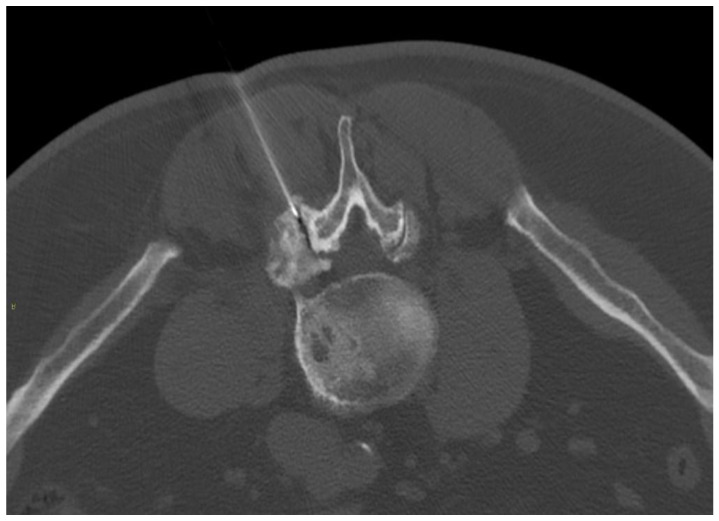
A 67-year-old male with severe lumbar facet joint arthritis underwent CT-guided L5-S1 right posterior joint corticosteroid injection.

The inferior process of the superior vertebrae and the superior process of the inferior vertebrae are the articular facets, which form a synovial articulation, also called a zygapophysial joint. In a prone position, a posterior approach can reach the joint capsule and puncture it with a needle (Figure 6), although a peri-articular injection may alleviate pain as well as an intra-articular injection [59].

**Figure 7:**
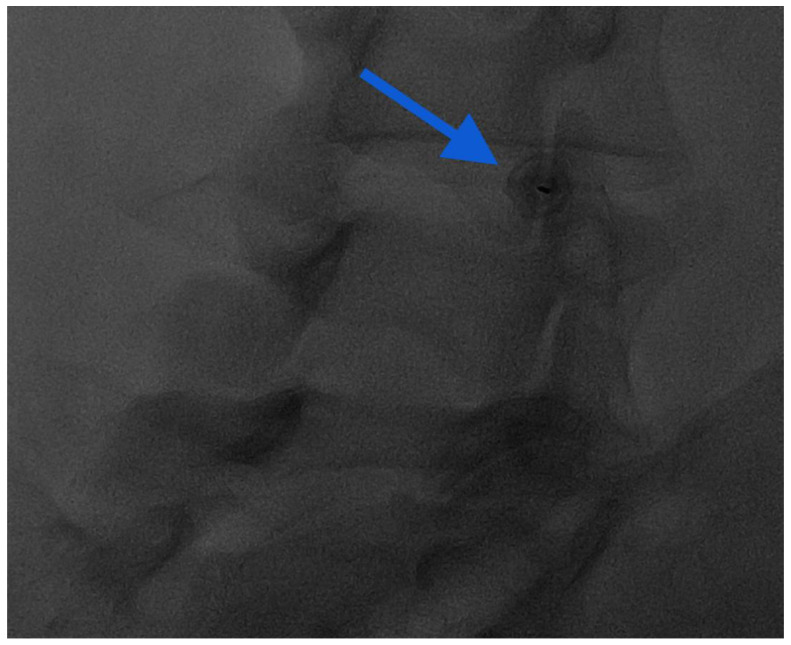
Oblique “Scotty dog” approach for intra-articular injection: notice the end-on view of the needle in the middle of the facet joint (blue arrow).

In addition to fluoroscopy (Figure 7) and CT, US can guide the procedure successfully. In a prone position, facet joints are evaluated by a convex probe with low frequency (3–8 MHz) and positioned 3–4 cm lateral to the spinous processes. Once the target has been found, the probe must be positioned in a transverse view in order to observe the articular structures. The puncture is performed by following the tip of the needle with the short side of the probe. Finally, the needle progresses with a lateral-to-medial trajectory towards the final target, which is the hypoechoic space center between the articular surfaces [58,60].

### 2.3. First-Level Image-Guided Procedures (Drug Injections)

The most frequent IR procedure to treat LBP is an image-guided drug injection. This offers targeted relief for patients meeting specific criteria, typically those with persistent pain despite conservative management or those unsuitable for surgery [48]. Patient selection relies on meticulous assessment, including detailed medical history, physical examination, and diagnostic imaging to pinpoint the source of pain accurately. Prior to treatment, traditional (X-ray) and advanced (CT and MRI) diagnostic modalities are indispensable for determining the most appropriate therapeutic approach for patients. CT and MRI are particularly valuable as they allow a complete examination of bone and soft tissue structures, facilitating the identification of potential causes of pain, and thus enabling the choice of the optimal therapeutic strategy. Despite the critical role of pre-treatment imaging in appropriate patient selection, there are limitations in correlating structural abnormalities with clinical symptoms [61,62]. In some cases, imaging findings do not correlate with the pain reported by the patient. Therefore, it is essential that physicians performing these procedures conduct a thorough clinical evaluation, which should include assessment of neurological deficits, evaluation of radicular symptoms, and identification of possible contraindications.

Under fluoroscopy, CT, or US guidance, the injection target is reached with a percutaneous approach. Fluoroscopy or CT guidance tends to increase the procedure’s success rate and minimize negative outcomes. Meanwhile, the US technique has the added advantages of no radiation exposure, distinguishing neural and vascular structures, and real-time visualization of the needle trajectory [44,59]. Then, medications are administered into the intervertebral discs (ID), FJ, or neural foramina (NF) in order to achieve pain resolution/reduction or local inflammation reduction [63].

Approach selection of the access site and determination of the appropriate needle path is made during pre-procedural imaging review [58]. Under fluoroscopy, the operator must modify the C-arm’s location in order to guide and insert needles, seen as thin radiopaque lines projected throughout the disc region, FJ, or over the intra-foraminal space [64]. With CT, a scan is acquired to evaluate the correct puncture site; subsequently, once the needle is inside, several CT scans can help the needle advancement monitoring until it reaches its target. US may guide the injections with the same efficacy, reducing X-ray exposure and helping to avoid vessels, in particular in intra-foraminal or intra-articular procedures [58,60]. 

#### 2.3.1. Injectable Materials

Several medications can be injected to reduce compression and inflammation, with minimal damage to the surrounding tissues. Steroids and methylene blue are some of the typical drugs that can attenuate the inflammatory response or eliminate the deteriorated disc by dehydration and breakdown of the nucleus pulposus [44,48,54].

A recent approach in intervertebral disc regeneration involves the insertion of biomaterials such as platelet-rich plasma (PRP), stem cells, and hydrogel. These new medications aim to repair and regenerate the disc by addressing the several disturbed pathways that underlie LBP [44,48].

##### Glucocorticoids

Glucocorticoid steroids, such as cortisone and prednisolone, are synthetic drugs that mimic the effects of cortisol, a hormone naturally produced by the adrenal glands. While primarily known for their anti-inflammatory properties, glucocorticoids also play a crucial role in regulating metabolism and immune responses. 

Glucocorticoids are one of the most used drugs in image-guided procedures. Inflammation is considered the main cause of LBP, and steroids (25 to 50 mg of prednisolone acetate) act to relieve patient pain and improve function by reducing the inflammatory response [65,66,67]. However, while effective for pain relief, there are potential side effects. These may include temporary discomfort at the injection site, flushing or redness of the skin, and a transient increase in pain (“steroid flare”). In some cases, individuals may experience allergic reactions, though these are rare. Prolonged or repeated use of glucocorticoid steroids can lead to more serious side effects, such as thinning of the skin, fat pad atrophy, skin depigmentation, weakening of nearby tendons or ligaments, and even joint infection. Additionally, systemic side effects may occur, including weight gain, increased blood sugar levels, mood changes, and suppression of the body’s natural production of cortisol [68,69]. Physicians should inform all patients with diabetes about the risks following glucocorticoid injection, including transient hyperglycemia. In fact, several studies have proven that steroids may induce the increase of glucose blood levels and hemoglobin A1C, even if the steroid dose and number of injections would not be expected to influence these effects [69,70,71]. 

For LBP, glucocorticoids can be injected into the FJ intradiscally and also intraforaminally. Ribeiro et al. [72] conducted a comparative analysis of the efficacy between intra-articular and systemic injections in a cohort of 60 patients diagnosed with FJS, revealing a marginal superiority of the intra-articular method. Several randomized controlled trials (RCTs) have proved the efficacy of steroid injections (either as monotherapy or in combination) within facet joints, demonstrating significant pain relief and functional enhancement. These outcomes are comparable to those achieved with other injectable substances [73,74,75]. 

Evidence from RCTs indicates that the administration of intradiscal glucocorticoids is superior in symptom alleviation compared with saline solutions or anesthetics (e.g., lidocaine) [66,67]. Even if prednisolone acetate and methyl-prednisolone acetate showed good results, in particular in patients with Modic 1 changes, they have an effectiveness limited to 1 month. Furthermore, at 3 months, treated patients reported paradoxically increasing pain. This behavior could be explained by both the short half-life and rebound effects of steroids [66]. Cao et al. demonstrated that intra-discal injection of steroids led to an improvement of pain scores at 3 or 6 months, rather than saline solution. However, this randomized control trial was disputed by several authors [66,67].

##### Methylene-Blue

Methylene blue (MB) was invented and synthesized in 1876 as a chemical dye. Its first use was for antimicrobial chemotherapy for malaria. Since then, it has been employed in several different fields of medicine, such as cancer chemotherapy, dementia, histopathology, and blood disinfection, thanks to its oxidation–reduction properties [76]. For instance, MB was widely used as a neurotropic drug for preventing damage to nerve terminals or blocking nerve conduction [77].

MB acts as a significant anti-inflammatory and antioxidant substance by upregulating the Nrf2/PRDX1 pathway, a scavenger of reactive oxygen species (ROS), and inhibiting nitric oxide synthesis. In addition, it denervates tiny nociceptive fibers that extend into the inner layer of the annulus fibrosis or nucleus pulposus, reducing pain in LBP sufferers [77,78]. 

Some recent meta-analyses affirm that intradiscal MB injection is a safe and effective minimally invasive approach for LBP. Nevertheless, the exact success rate is yet uncertain [77]. Other authors [79] found that the MB neurolytic activity helped to reduce pain in patients with chronic LBP (CLBP) by 30% in 40% of patients at 6 months. Kim et al. [80] affirmed that MB shows a short-term efficacy in reducing pain scores, with the maximum at 3 months. Nevertheless, a recent RCT [81] comparing MB injection with placebo injection demonstrated no significant differences between the two groups, and did not suggest using MB as a standard approach for CLBP management. 

##### Biologic Agents (Platelet-Rich Plasma, Mesenchymal Stem Cells, Hydrogel, and Hyaluronic Acid)

Although there is growing interest in intradiscal injections of biological substances such as platelet-rich plasma (PRP) or mesenchymal stem cells (MSCs), which are hypothesized to have regenerative capabilities, the data behind their use in clinical practice are not yet clear [82].

The first use of PRP was for the treatment of thrombocytopenia in the 1970s. Then, it was introduced as a treatment for musculoskeletal diseases due to its dual properties of inducting cell proliferation and inhibiting inflammatory pathways. In the last 30 years, PRP has been applied in several fields, and, in recent years, it has begun to be used in chronic orthopedic diseases [83,84]. Many techniques to prepare PRP are known, and so multiple different types of PRP can be produced. In general, autologous peripheral blood is centrifuged to create a concentrated platelet solution in which a high concentration of platelets and factors is reached, including growth factors, cytokines, chemokines, and other plasma proteins [85]. PRP has been successfully used in several trials on both human and animal subjects to treat disorders that need collagen-based tissue reparation, including treating tendons, ligaments, and cartilage that have been injured or that have deteriorated [83,85]. PRP has the potential to promote cell proliferation, differentiation, migration, and synthesis of extracellular matrix proteins through the blood supply [85]. In this way, it slows disc degeneration progression by sealing tissue breaks and repairing them, helping to prevent fluid leaks [83,84,85]. Even if the degenerated disc has a reduced blood supply, it has been demonstrated that an intradiscal PRP injection manages to reduce the inflammatory response because of its anti-inflammatory abilities by inhibiting several pathways such as the Bcl-2-associated death promoter, which triggers apoptosis, and glycogen synthase kinase-3β, which encourages the breakdown of β-catenin [84,85].

MSCs are multipotent adult stem cells that have the ability to proliferate and differentiate into a variety of cell lineages, including cells that are within the nucleus pulposus and the intervertebral discs [86,87]. In recent decades, MSCs have been extensively used in regenerative medicine, including musculoskeletal diseases. These biological agents present excellent accessibility since they can be quickly and safely separated from different tissues, especially from the bone marrow and adipose tissue [86]. One of the underlying mechanisms consists in activating local nucleus pulposus (NP) cells by releasing growth factors, which will cause MSCs to differentiate into NP cells. This activation might stimulate the production of essential elements of the extracellular matrix, regenerating the disc, even if a “restitution ad integrum” is not reachable [86]. Additionally, MSCs may attenuate the inflammatory response in the disc by encouraging the synthesis of anti-inflammatory factors due to their strong immunomodulatory abilities [32,44,59,60,86,87].

Some studies and some meta-analyses have shown a positive correlation between reduction in pain and intradiscal injection of PRP or MSCs. Some authors suggest that these biological agents manage to both reduce symptoms and improve functional scores [88,89,90,91,92,93]. Nevertheless, a recent meta-analysis and two reviews focusing on PRP and MSCs confirmed that the evidence base for intradiscal PRP or MSCs for the treatment of chronic LBP is of very low quality overall. In fact, there were no clear certainties about injectate composition, preparation, or patient eligibility requirements, and clinical outcomes are controversial [82,94,95].

Recently, a number of hydrogel-based materials have been created to improve the efficiency of MSCs. The injection of MSCs is intended to encourage the differentiation of NP cells in order to restore the structure and function of the disc; nevertheless, these cells need an optimal microenvironment in order to proliferate and thrive. Adding hydrogel materials, such as hyaluronic acid-based hydrogels, might repair the extracellular matrix (type II collagen and proteoglycans), hence restoring the mechanical properties of the disc and boosting the activity of NP cells. Moreover, they could have anti-inflammation and anti-nociceptive proprieties [96]. In the literature, most of the studies have been conducted in vitro or on animals, although two studies on humans, a first phase clinical trial and an RCT, showed improved results and efficacy of a combined injection of MSCs and natural hydrogels [97,98].

Hyaluronic acid injection has also been used for facet joint osteoarthritis. Fuchs et al. [99] compared hyaluronic acid injection with glucocorticosteroids, and found significant pain relief, improving quality of life and function, with both treatments. On the other hand, Annaswamy et al. [73] found in their study a better long-term improvement with hyaluronic acid, with similar benefits with both molecules on a short-term basis.

In Table 2, we provide a summary of the characteristics, advantages, and disadvantages of the main available injectables, drugs, and materials.

### 2.4. Second-Level Image-Guided Procedures

#### 2.4.1. Oxygen–Ozone Therapy

Ozone is a strong oxidizing gas that is present in the atmosphere, but it can also be produced artificially. The chemical properties of the oxygen–ozone (O2-O3) combination cause several biochemical effects in the human body, including anti-inflammatory activities [101].

**Figure 8:**
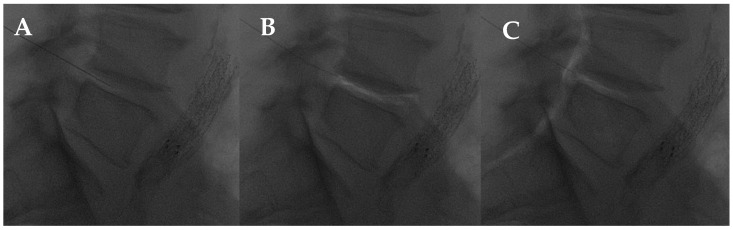
L4-L5 intradiscal oxygen–ozone chemonucleolysis. (**A**) Intradiscal puncture on lateral view; (**B**) intradiscal; and (**C**) intraforaminal oxygen–ozone mixture injection.

Essentially, percutaneous O2-O3 treatment is the main application for musculoskeletal disorders, such as arthritis, tendonitis, or other diseases, including chronic LBP [102]. The mechanisms of action of the intradiscal, facet joint, and periganglionic O2-O3 injection have been explored in many studies. In the interaction with water and polyunsaturated fatty acids, O2-O3 generates reactive oxygen species (ROS), which results in the cascade of antioxidant response elements and the subsequent downregulation of the inflammatory response. Several enzymes are involved in this process, including nuclear factor-erythroid 2-related factor 2 (NRF2), a key element for antioxidant human mechanisms [101,103,104]. The capacity of the nucleus pulposus to hold water may be compromised by the O2-O3 oxidizing activity, which may disrupt glycosaminoglycan chains and dehydrate the nucleus pulposus. The O2-O3 chemodiscolysis would reduce the size of the hernia and eliminate hernial conflict (Figure 8), despite not being able to alter the natural course of the disc degeneration process and endplate changes (Modic changes) [105,106,107]. 

**Figure 9:**
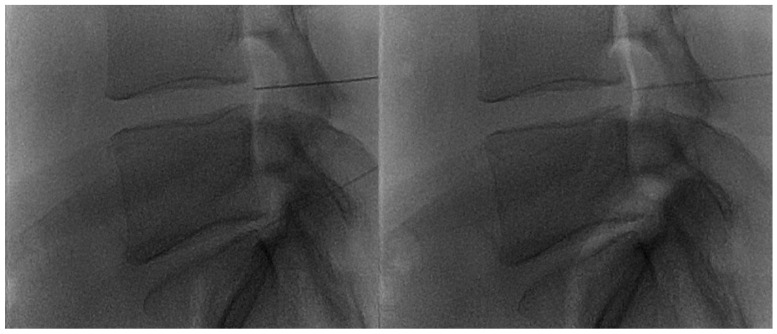
Lateral fluoroscopic views obtained after intraforaminal oxygen–ozone mixture injection: notice the propagation of the oxygen–ozone along the epidural space (“white” opacity).

The process of dehydration and volume reduction might also be accelerated by concurrent periganglionic O2-O3 infiltration near the disc (Figure 9). Thanks to its strong tissue diffusion ability, O2-O3 is able to work as an anti-inflammatory and anti-edema molecule, as well as an analgesic locally on the inflamed ganglion root, producing more clinical advantages and a successful outcome. In addition, oxidizing algogenic receptors of nociceptive roots that develop around a fractured disc would block the pain signal and, consequently, favor muscle relaxation [102,104]. In addition, O2-O3 promotes pain relief by downregulating the inflammatory response through the prostaglandin pathways and initiating the disc repair process by boosting fibroblast activity, including collagen deposition and chondrocyte hyperplasia [101]. Overall, these mechanisms contribute to reducing pain [101,103,104]. 

**Figure 10:**
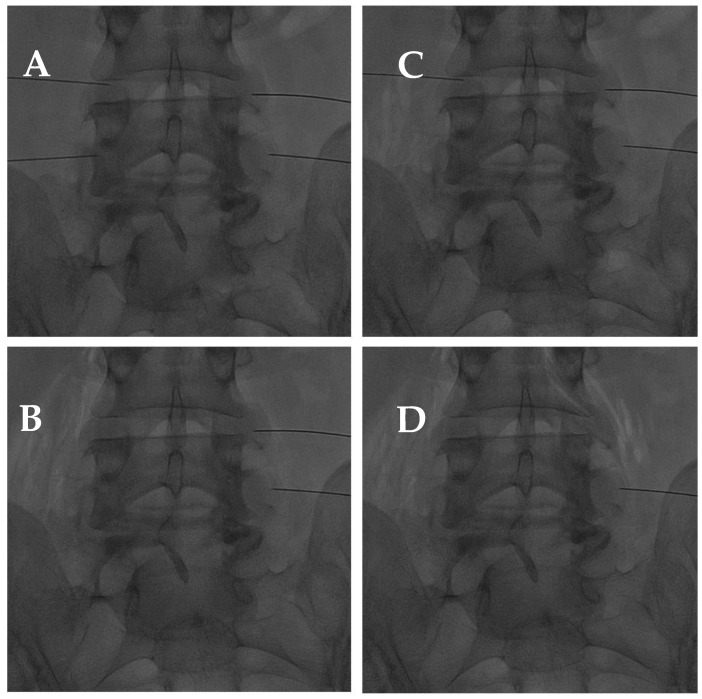
(**A**) AP fluoroscopic view of four bilateral facet joint puncture; and (**B**–**D**) oxygen–ozone mixture intra-articular injection: notice the propagation of oxygen–ozone along the psoas muscle fibers due to pulling the needle back under high pressure.

In addition, high levels of ROS have been found in progressed facet joint degeneration since they induce apoptosis pathways, in particular in chondrocyte cells [108]. Hence, in reducing the inflammatory response and oxidative stress, O2-O3 demonstrated good efficacy as anti-antalgic therapy in CLBP, even caused by facet joint syndrome (Figure 10) [60]. Even if the O2-O3 mixture is widely used for these musculoskeletal indications, there is a low level of evidence for its administration, except for CLBP or knee osteoarthritis [102]. 

The infiltration of O2-O3 should be delivered at a concentration between 1 and 40 μg per milliliter of oxygen in order to minimize toxicity and achieve optimal outcomes in percutaneous injections [103]. Reaching 50 μg/mL could cause iatrogenic injuries on the ring [102]. In addition, O2-O3 intradiscal injection is often paired with paraganglion or intraforaminal injection of other substances, including steroids, anesthetics, or ozone itself.

Patient selection is crucial to avoid failure and reach optimal outcomes. For intradiscal injections, cauda equina syndrome or motor deficits, infections, fractures, malignancies, herniated calcified discs, free disc fragments, and extruded hernia should be considered exclusion criteria, but insufficient evidence exists to provide these recommendations [44,109,110,111]. Moreover, during clinical examination, the patient should describe discomfort in terms of the specific dermatome involved. No limit of age and no gender differences have been found in the literature, but better results are reached in younger people with a single herniated disc [110].

In CLBP management, the O2-O3 image-guided injection may be performed from 3 to 10 times (often one or two a week), depending on the clinical progression of the patient. Cases not responding after two or three attempts are considered unsuccessful [102].

Considering just intraforaminal infiltration, an RCT by Bonetti et al. [112] demonstrated that ozone injections are more effective than steroid peri-radicular injections for lowering CLBP. In particular, they found significantly better outcomes in patients with disc disease.

Andreula et al. [113], compared an image-guided injection of O2-O3 alone to O2-O3 intradiscal injection + steroids and anesthetic paraganglion injection, and found good outcomes in both procedures, with better results in the latter group (70% and 78%, respectively, *p* ≤ 0.05). In contrast, a recent RCT found that any further periforaminal steroid injection is not more effective than O2-O3 injection alone [114].

Some authors compared the usage of intradiscal and paraganglion injections of O2-O3 with the same treatment, adding corticosteroid, not finding any statistical differences between the groups, but reaching the same success rate as described before. In addition, excellent outcomes have been reported, even at long-term observation (12 months) [63,111,115,116].

Buric et al. [106] have also used long-term follow-up (5 and 10 years), producing results that are consistent with the other literature (>80%). Additionally, they compared the MR characteristics of the disc after surgery or radiofrequency ablation with those following O2-O3 intradiscal injection, describing the same dehydrated aspects in each group in the long follow-up.

A recent RCT compared three different treatments. The results suggest that the number of people who required surgery after the first treatment was lower in the group that received the O2-O3 injection than in the group treated with oxygen alone (20% vs. 11%). Despite the small number of patients, the authors affirm that intradiscal O2-O3 therapy can reduce the need for surgery [117].

Kelekis et al. [118] also conducted an RCT comparing O2-O3 injection and surgery. In terms of clinical outcomes after six months of follow-up, they reported that ozone and surgical treatment provided identical results. In addition, 71% of patients who received O2-O3 treatment were able to avoid surgery.

Overall, the protocols for O2-O3 image-guided injections are poorly standardized, making it difficult to evaluate various research in systematic reviews and provide recommendations of the highest caliber. Despite this, some reviews and meta-analyses that specifically address the use of O2-O3 for CLBP in the cervical and lumbar zones have been published. In general, it has been proven that intra-disc injection of O2-O3 is a minimally invasive, safe, and effective therapy for relieving pain caused by a herniated disc, improving function in the short (<6 months) and medium term (>6 months), with an estimated total complication rate of less than 0.1% [109,119].

#### 2.4.2. Image-Guided Radiofrequency

For more than 50 years now, radiofrequency (RF) has been applied to a variety of medical conditions. It is usually used to treat pain through thermal lesions and fiber destruction by conducting continuous nociceptive input via the percutaneous route [120,121].

Some studies have reported the neuro-modulatory and anti-inflammatory effects of radiofrequency. Microscopic damage is observed in membrane abnormalities and mitochondrial morphology, as well as through interruption and disorganization of microfilaments and microtubules. This ultrastructural pathway occurs more widely in type C and type A nerve fibers, which are the main nociceptors. Furthermore, radio waves act on immune cells, inhibiting proinflammatory cytokine production, such as interleukin-1b and interleukin-6 [122,123,124].

Nowadays, radiofrequency therapy is the medical procedure mainly used to reduce LBP, with a low complication rate (less than 1%), ease of application, and low cost [122,125,126].

Treatments are divided into continuous RF (CRF) and pulsed RF (PRF) using an electromagnetic field [120]. These procedures are principally executed with CT guidance or fluoroscopy guidance. Even if MRI guidance is not employed in daily practice yet, there are some studies that support its use, especially in those patients affected by degenerative enlarged facet portions that can make it difficult to see, with the radiations, the course of the medial dorsal ramus [127].

CRF is the standard for the thermocoagulation of the dorsal ramus medial branch for managing FJS, whereas PRF is used for pain trigger points, painful joints, peripheral neuropathies, and chronic dorsal root ganglion (DRG) radiculopathies [128,129].

##### Continuous Radiofrequency

In the treatment of FJS, each lumbar facet joint receives innervation from the medial branches of the dorsal rami, and CRF stimulation leads to a temperature increase of these nerve fibers, damaging them above 45 °C. The result is nonselective damage of the myelinated and unmyelinated nerves. Ablation is the result of heat dissipation from the needle catheter, generally with a 22-gauge cannula and 5 mm tip length, for 90 second (s) at a temperature of 80 °C [130].

At the L1–L4 levels, the medial branch bears a constant relationship to the bone, where it runs across the root of the superior articular process, and then an appropriate target point is the dorsal surface of the root of the transverse process immediately below the most medial end of its superior edge. At the L5 level, the medial branch is not suitable for percutaneous radiofrequency neurotomy. At the L5 level, the dorsal ramus is the target. The target point for this nerve is where it runs along the groove between the ala of the sacrum and the root of the superior articular process [131].

Two studies have assessed the efficacy of CRF ablation for treating sacroiliac pain, both of them using continuous, cooled RFA procedures. These studies used a 17-gauge cannula and a 4 mm tip length. In particular, Cohen et al. heated the device to 80 °C for 90 s [132]; on the other hand, Patel et al. used 60 °C for 150 s [133]. These two studies assessing the efficacy of RFA for treating sacroiliac joint pain found a statistically significant reduction in pain for the intervention group when compared with the control group [130,132,133].

##### Pulsed Radiofrequency

PRF consists of a high-intensity electromagnetic current delivered in pulses, which allows heat to dissipate during the latent period so that neurodestructive temperatures cannot be reached. It is used especially for the treatment of discogenic pain (DP), but it can also be used for FJS [131].

PRF has a different effect mechanism based on a combination of other neurobiological effects. Erdine et al. evaluated ultrastructural lesions on sensory nociceptive axons occurring after PRF intervention. They affirmed that PRF action selectively produced a wider range of lesions in smaller primary sensory nociceptors, such as Aδ and C fibers, compared with larger non-pain sensory fibers. PRF activates the descending noradrenergic and serotoninergic pain inhibition pathways and inhibits excitatory nociceptive C fibers [134]. PRF is the procedure of choice for DP, compared with CRF, based on its safety profile, even if research results regarding PRF’s effectiveness as a modality of pain therapy are mixed [123].

**Figure 11:**
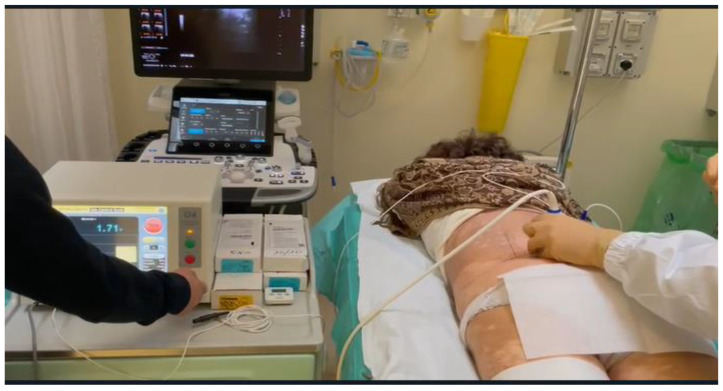
Ultrasound-guided pulsed radiofrequency treatment of L5 dorsal roots ganglion in a 75-year-old female patient with persistent low back pain and chronic sciatica.

**Figure 12:**
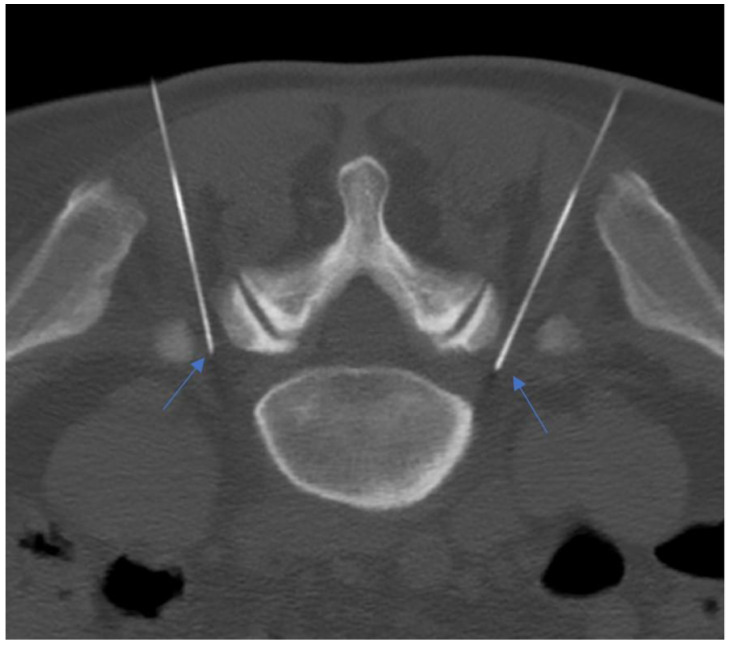
A 55-year-old patient with lumbosacral discogenic pain treated with bilateral pulsed radiofrequency; the needles are located at around 5 mm close to the L5 ganglion bilaterally (blue arrows).

PRF uses intermitted administration of a high-frequency current, allowing heat to disperse to the surrounding tissues, avoiding a temperature rise over the critical level of 42 °C. Typically, RF current (50.000 Hz) is applied in 20 ms pulses, at a frequency of 2 per second for 120 s. A 20–22 G needle electrode with an active tip is introduced and advanced toward the target DRG. The RF current should be activated only if imaging results are confirmed by the exact positioning of the needle on the target symptomatic DRG (Figure 11 and Figure 12) [135,136].

Marliana et al. determined that the PRF effect was not significant in reducing radicular pain scores due to lumbar DP compared with controls four and eight weeks after the treatment. However, PRF had a significant effect in lowering the radicular pain score 12 weeks after the treatment. PRF is relatively safe and has minimal side effects [122].

Teixeira and Sluijter assessed the application of intradiscal PRF (ID-PRF) for the treatment of discogenic LBP. This study has suggested that percutaneous ID-PRF may reduce nociceptive input from the intervertebral disc. In addition, some studies reported beneficial effects of ID-PRF on discogenic LBP. For discogenic pain, a high-voltage and long-duration PRF was recommended, with a duration of 15 to 20 min [137].

Some studies support PRF in the management of FJS, defining it as a promising technique, even if its effectiveness is weaker compared with CRF. PRF could be considered as an alternative and should be used in clinical practice in selected patients’ treatment because of its advantages over CRF. In fact, PRF is safer and reduces the risk of tissue damage. Patients also reported a very high level of satisfaction, demonstrating that a less-invasive approach like PRF could be a better option for some patients [120,124,131,137,138].

#### 2.4.3. Magnetic Resonance Imaging (MRI)-Guided Focused Ultrasound

Magnetic resonance imaging (MRI)-guided focused ultrasound (MRgFUS) is a minimally invasive thermal treatment modality that uses a phased-array ultrasound transducer embedded inside the MRgFUS patient table integrated with the magnetic resonance imaging (MRI) scanner. During MRgFUS treatment, ultrasound (US) energy is selectively focused within target tissues, causing localized thermal ablation. MRI is used for treatment planning, the guidance of the US beam, real-time magnetic resonance (MR) thermometry, and for treatment assessment. MRgFUS is used to treat various tumors, neuropathic pain, and painful bone metastasis. Additionally, multiple research studies have focused on the ability of FUS to disrupt nerve conduction and cause necrosis of nerves, including MRgFUS renal sympathetic denervation and ablation of sciatic and intercostal nerves [139]. In our context, the only therapeutic indication for MRgFUS is the treatment of FJS, and, to develop its clinical application, two recent studies demonstrated the safety and feasibility of MRgFUS ablation in preclinical [140] and clinical [141] settings.

Weeks et al. [141] reported a reduction in average and worst pain, respectively. They used a clinical FUS system installed in a 3 T MRI scanner. The default values of sonication duration, 20 s, and cooling time were automatically calculated by the treatment planning software, and were 90 s or longer. The treatment was monitored in near real-time using the rapid acquisition of MR images, from which temperature maps were calculated by the planning software (InSightec) based on the proton resonance frequency shift (PRFS) baseline subtraction approach. The procedure was conducted by directing the FUS beam to the facet joint itself, and aimed to achieve denervation by ablating the periarticular tissue and the nerves inside it, rather than at the MB nerve (as is commonly done during RF ablation), to avoid interaction of the FUS beam with critical structures such as the spinal cord and the nerve root.

An alternative targeting strategy is to adopt the approach used in the treatments with radiofrequency (RF) and aim the FUS beam directly at the MB nerve. The feasibility of such an approach can be hypothesized based on the acoustic properties of bone and clinical experience with RF ablation in the spine. Due to its high density and heterogeneous composition, bone tissue reflects and attenuates ultrasound at a much greater rate than muscle. This characteristic and the presence of spinal fluid and the venous plexus play an important role in protecting the spinal cord and adjacent nerve roots [142,143,144].

In Table 3 we provided a comparison of main advantages, disadvantages, and indications of second-level procedures.

## 3. Future Directions and Limitations

Looking ahead, the future of image-guided procedures for low back pain holds significant promise with the continued advancement of technology and techniques. One key direction lies in the refinement and integration of artificial intelligence (AI) algorithms into image guidance systems. AI can enhance preoperative planning by analyzing vast amounts of patient data to personalize treatment strategies and predict surgical outcomes more accurately. Moreover, real-time intraoperative navigation systems are likely to become more sophisticated, incorporating AI-based decision support tools to assist surgeons in precisely targeting spinal structures, minimizing surgical trauma, and optimizing implant placement. Concurrently, the development of novel imaging modalities, such as high-resolution 3D reconstructions and intraoperative MRI, will further enhance visualization and navigation accuracy, enabling surgeons to perform minimally invasive procedures with unprecedented precision.

Recently, the combination of different image-guided techniques at the same time in order to enhance the therapeutic effect has become an interesting topic. Napoli et al., in a recent randomized clinical trial, evaluated the combination of a corticosteroid injection and pulsed radiofrequency vs. steroid injection alone in patients with discogenic lumbar pain. They found that a synergistic combination of both treatments is more efficient in relieving symptoms compared with a single corticosteroid injection [145]. This suggests that the contemporaneous use of first- and second-level procedures could be a safe and effective possibility in patients with LBP.

Even though lumbar spinal canal stenosis is considered an indication for surgery, image-guided percutaneous interspinous process device (ID) insertion for canal dilatation has been suggested as a treatment option and could be considered when surgery is not indicated [146]. Phan et al., in their systematic review and meta-analysis comparing ID versus traditional decompression, concluded non-inferiority in terms of VAS pain scores after the procedure, with a lower complication rate with ID [147]. Nevertheless, another systematic review and meta-analysis concluded a big limitation is an increased reoperation rate [148].

In the last 10 years, posterior face joint fixation by performing percutaneous approaches has also been proposed in order to reach an immediate stabilization of the lumbar spine, almost similar to the surgical approach. Percutaneous vertebral fixation with screws is considered the last resort of percutaneous treatment for degenerative lumbar spine disorders [149]. The use of screws in percutaneous vertebral fixation provides immediate spinal stability, reduces pain and disability, and allows early mobilization. Additionally, percutaneous vertebral fixation is a minimally invasive procedure, which leads to lower blood loss, shorter hospital stays, and faster recovery. Marcia et al. [150] reported that, in lumbar pain caused by high-level disc degeneration combined with facet joint hypertrophy and canal stenosis, percutaneous pedicle screw fixation led to a significant reduction of VAS pain scores and a significant improvement in the Oswestry disability index (ODI) after 1 month and after 1 year. Another study by Amoretti et al. [151] demonstrated that percutaneous pedicle screw fixation is a safe procedure that has an excellent or good success rate in clinical terms and requires less than one hour.

Technological improvements and increased skills of radiologists foreshadow an increase in the number of treatments available for every patient. Despite the increasing popularity of image-guided procedures for low back pain, significant limitations persist due to the lack of robust scientific evidence supporting their efficacy and long-term outcomes. For instance, there are some studies that did not find more effectiveness compared with placebo, in particular, for facet joint injections [72,152,153]. Furthermore, there is still a lack of sufficient scientific evidence regarding some image-guided procedures; a good example is radiofrequency denervation for CLBP. Maas et al., in their systematic review, found only low-quality evidence for the use of this procedure for CLBP, concluding that multi-institutional studies with larger sample sizes are still needed [154].

Additionally, the reliance on imaging modalities like fluoroscopy, CT, or MRI poses constraints due to radiation exposure, feasibility issues, and interpretation challenges. Further well-designed randomized controlled trials and long-term observational studies are imperative to address these knowledge gaps and guide evidence-based clinical decision-making in the management of low back pain.

## 4. Conclusions

Chronic low back pain is a significant problem in developed countries, mostly related to its high prevalence and economic burden. Therefore, it is imperative to find multiple low-cost and minimally invasive treatment possibilities that could change disease outcomes in patients where conservative treatment is not effective, and surgery is not indicated or relatively contraindicated.

Although, for some types of image-guided procedures, there is still a need for stronger scientific evidence, it has been proven that most procedures are effective in reducing symptoms and improving quality of life. Interventional radiology has a pivotal role in reducing the necessity of surgical procedures and drug intake.

In this context, the application of the ‘Interventional Radiology Outpatient Clinics’ may be of great help, allowing radiologists to merge clinical and imaging data and to discuss directly with patients the possible role of the myriad of minimally invasive interventional procedures [155].

## Figures and Tables

**Table 1:** Summary of the different imaging modalities with their advantages/disadvantages, and their main indications.

Imaging Guidance	Advantages	Disadvantages	Uses
Ultrasound	- No Radiation exposure - Low cost - High availability - Dynamic imaging	- Operator dependent - Less spatial resolution for deep structures - Difficult in obese patients	- Most common: Facet Joint Injections - Less common: Intraforaminal injections
MRI	- No radiation exposure - Good spatial resolution - 3D imaging	- Very expensive - Time consuming	- Most common: FUS - Less common: Radiofrequency, Injections
CT	- Good availability - Optimal spatial resolution - 3D imaging	- Radiation exposure - Relatively expensive	- Injections - Radiofrequency - Ozone therapy
Fluoroscopy	- Dynamic imaging - Good availability	- Radiation exposure - Lack of contrast resolution (disc and neural roots not visible)	- Ozone therapy - Injections

**Table 2:** Comparison of the different injectable drugs/materials that can be used in the management of CLBP.

Drug/Material	Advantages	Disadvantages	Indications
Glucocorticoids [66,67]	Anti-inflammatory effect Most used Cheap Rare side effects	No long-term effect Possible rebound effect	FJS DDD Radicular pain
Methylene Blue [76,77,78,79,80,81,100]	Double action: anti-inflammatory and antioxidant effect	Uncertain success rate No long-term effect	DDD Radicular pain
Platelet-rich plasma (PRP) [82,83,84,85]	Triple action: anti-inflammatory effect and induction of cell proliferation, differentiation, migration, and the synthesis of extracellular matrix proteins Several different types of PRP can be produced	Unclear data for clinical practice due to lack of high-quality studies No clear indications for composition, preparation, or patient eligibility	DDD Radicular pain
Mesenchymal Stem cells (MSCs) [86,87]	Double action: anti-inflammatory effect and stimulation of NP cells (regenerating extracellular matrix) Easy to produce Low complication rate Possible combination with hydrogels	Lack of high-quality studies No clear indications for composition, preparation, or patient eligibility	DDD Radicular pain
Hydrogel [96]	Stimulation of NP cells by providing a good microenvironment Cheap Low level of toxicity Possible combination with MSCs reducing side effects and long-term positive effects	Never alone Lack of high-quality studies	DDD Radicular pain

**Table 3:** Comparison of second-level techniques with their advantages, disadvantages, and main indications.

Second Level Procedures	Advantages	Disadvantages	Main Indication
Radiofrequency	Relatively less expensive; Can be US-guided, MR or CT guided	Non-target ablation: skin burns	Continuous: Facet joint OA Pulsed: Discogenic pain
Ozone therapy	Can be a curable option	Higher rate of complications in inexperienced operators; the necessity of CT or MR guidance	Discogenic pain
MRgFUS	Non-invasive option with optimal real-time monitoring	High cost and long procedure with inexperienced operators	Facet joint OA

## Data Availability

For details, data, and further information contact the corresponding author.
